# Measuring the burden of infodemics on health outcomes through harmonized global metrics

**DOI:** 10.1093/eurpub/ckac131.326

**Published:** 2022-10-25

**Authors:** A Ishizumi, AG Dunn, T Purnat, B Yau, C Bertrand-Ferrandis, B White, S Briand, T Nguyen

**Affiliations:** 1 Epidemic & Pandemic Preparedness & Prevention, WHO, Geneva, Switzerland; 2 Biomedical Informatics and Digital Health, University of Sydney, Sydney, Australia

## Abstract

**Issue/problem:**

Infodemics happen when an excess of information makes it difficult for people to discern what they see and hear to make good health decisions. Several challenges limit the usefulness of applying infodemiology research to the practice of managing infodemics including inconsistency in how information exposure is measured and a lack of focus on assessing associations with health behaviors.

**Description of the problem:**

In 2021, WHO partnered with the University of Sydney to develop a study toolkit. We sought to create novel tools for measuring information exposure that can be easily deployed, linked to surveys measuring health behaviors, and implements a standardized study protocol so that data can be directly synthesized into a global analysis of information risk factors associated with health behaviors.

**Results:**

A web-based study platform was developed, comprising tools for capturing information exposures within studies that link to health behavior surveys. The first tool is a smartphone application that asks users to actively record relevant information they see or hear in diary. The second application is a web browser plugin that passively tracks webpages with relevant keywords. Because localized studies follow a standardized protocol and de-identified participant data are recorded in a common format, local study investigators can opt-in to contributing study data to support global surveillance efforts.

**Lessons:**

Through standardization of measurement tools and relevant study protocols, the toolkit can be used to quickly collect and synthesize data for global or regional analysis of infodemics, including in Europe. Validation of the toolkit in the field is needed to inform its open-source release.

**Key messages:**

• A toolkit for measuring information risk factors associated with behavioral outcomes was developed.

• Global collaboration using the toolkit can improve synthesisability of studies investigating infodemic burden of disease.

